# Iron homeostasis in *Mycobacterium tuberculosis* is essential for persistence

**DOI:** 10.1038/s41598-018-35012-3

**Published:** 2018-11-26

**Authors:** Manitosh Pandey, Sakshi Talwar, Sutapa Bose, Amit Kumar Pandey

**Affiliations:** 1 Mycobacterial Pathogenesis Laboratory, Translational Health Science and Technology Institute (THSTI), Faridabad, 121001 Haryana India; 2 Earth and Environmental Science Research Laboratory, Dept. of Earth Sciences, Indian Institute of Science Education and Research, Kolkata, Mohanpur, Nadia, West Bengal India

## Abstract

Tuberculosis, caused by the obligate intracellular pathogen *Mycobacterium tuberculosis* (Mtb), is responsible for 2–3 million deaths annually worldwide. Intracellular adaptability, which is critical for long-term persistence, requires the pathogen to neutralize host-mediated insults. The iron–sulphur (Fe–S) cofactor is essential for many enzymes critical for such ‘adaptation’. The Mtb genome harbors only one putative iron–sulphur cluster (ISC) operon (*rv1460-66*) predicted to be involved in the generation of the Fe–S cofactor. Except for *rv1460*, all other genes in this operon are anticipated to be essential. The current study investigated the role of *rv1460*, an *sufR* homologue of Mtb (*sufR*_*TB*_), in maintaining intracellular Fe homeostasis and its implications on mycobacterial pathogenesis. We found that Mtb ISC locus (*rv1461–66*) was transcribed as a single multigene transcript. We successfully generated the *sufR*_*TB*_ null mutant strain (*ΔsufR*_*TB*_) of Mtb, suggesting nonessentiality of the gene under normal growth conditions. The mutant strain demonstrated enhanced biofilm generation and failed to grow under a low-Fe condition. Growth characterization studies indicated that SufR_TB_-mediated intracellular Fe homeostasis is essential for Mtb to persist in the host. Targeting mycobacterial persistence by inhibiting SufR_TB_ protein activity may be a novel intervention strategy in tuberculosis treatment.

## Introduction

The iron–sulphur (Fe–S) cluster, which is the most primitive, versatile, and essential prosthetic group, is required by all organisms, including bacteria, for their survival. Crucial cellular processes, such as electron transfer (respiration, ferredoxins, and hydrogenases), enzyme catalysis (central metabolism and DNA replication and repair), and gene expression regulation (transcription factors sensing oxygen, Fe, and oxidative and nitrosative stress), are all dependent on the Fe–S cluster as a cofactor^1–3^. In addition, scavenging of free soluble Fe during the Fe–S cluster biogenesis process protects organisms from the deleterious effects of Fe^1^. Three systems involved in the biogenesis of the Fe–S cluster in bacteria have been identified: the nitrogen fixation system, the iron–sulphur cluster (ISC) system, and the sulphur mobilization (SUF) system^4–6^. Although the distribution, number, and arrangement of these systems vary among species, functionally, these proteins assemble and transport the Fe–S cluster to apoproteins^7^.

Unlike in other bacteria, the presence of the ISC operon as the only Fe–S cluster biogenesis system in *Mycobacterium tuberculosis* (Mtb) renders the entire operon essential for the growth of Mtb^8^. The ISC operon is upregulated under low Fe and stress conditions (both oxidative and nitrosative) and during the growth of Mtb in macrophages^9,10^. The entire seven-gene operon, with the exception of *sufR*_*TB*_ (*rv1460*), is essential for the growth of Mtb^11^. Although the significance of Fe in mycobacterial pathogenesis is well-documented^12–14^, the role of SufR_TB_ protein in regulating intracellular Fe homeostasis and Fe–S cluster biogenesis, as well as its effects on mycobacterial pathogenesis, have never been studied. Recently, the SufR_TB_ protein of Mtb was reported to negatively regulate the expression of downstream ISC genes^15^. The same study suggested that the absence of sufR gene is deleterious for Mtb and that this transcription repressor is not required for growth under a low-Fe condition^15^.

Mtb residing in an Fe-deficient milieu triggers an extensive response aimed at acquiring Fe from the host. This response is critically regulated by Mtb to prevent the accumulation of toxic levels of Fe inside the cell. Mtb regulated this by potentially modulating the transcription levels of genes involved in the sensing, uptake, transportation, and storage of Fe^12,16–19^. We hypothesize that by modulating the expression of Fe-mobilizing ISC genes, SufR_TB_ protein regulates Fe homeostasis in Mtb under various growth and stress conditions. In the current study, we aimed to understand the regulation of the ISC locus by *sufR*_*TB*_ gene and its implications on mycobacterial pathogenesis. We found that the ISC gene locus is a seven-gene operon transcribed as a single gene transcript. We successfully generated *sufR*_*TB*_ null mutant strains, thus establishing the nonessentiality of this gene under *in vitro* growth conditions. Concurrent with the finding of a previous study^15^, we observed that the absence of SufR_TB_ protein caused an increase in the transcript levels of ISC genes. In addition, we demonstrated that *sufR*_*TB*_ gene is essential for the growth of Mtb under low-Fe and stress conditions. The mutant strain demonstrated an enhanced biofilm generation phenotype. Furthermore, excess accumulation of intracellular Fe in the *rv1460* mutant strain underscores the role of SufR_TB_ protein in Fe sensing and homeostasis. Finally, this putative transcription factor was found to be essential for the *ex vivo* growth of Mtb in mouse bone marrow-derived macrophages (BMDMs) and for the *in vivo* growth of Mtb in a mouse tuberculosis infection model.

## Results

### Generation of the *sufR*_*TB*_ gene deletion mutant strain in Mtb

**Figure 1:**
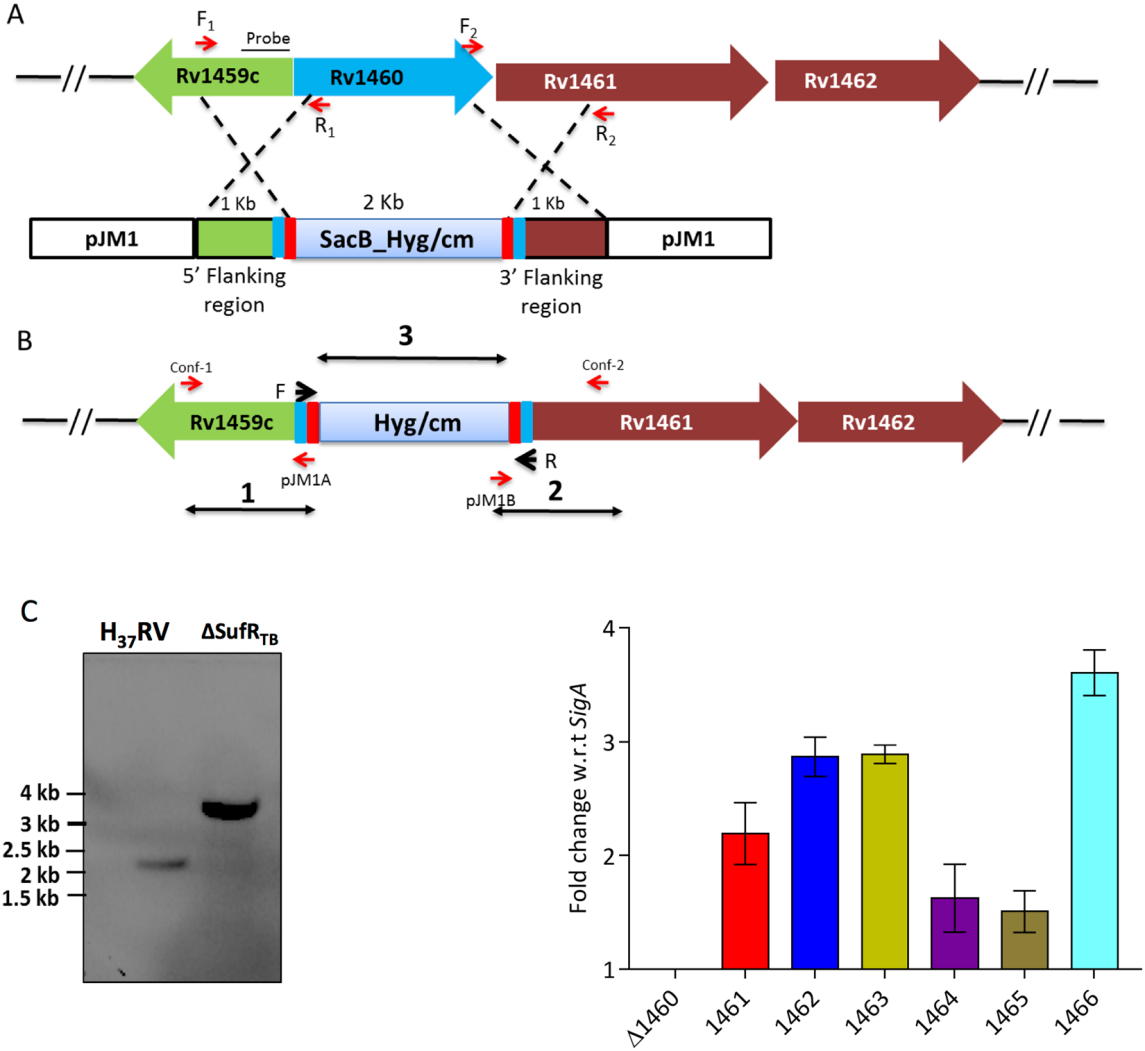
Generation of Mtb *sufR* deletion mutant. **(A**) Schematic representation of the homologous recombination between the upstream and downstream region of Rv1460 gene cloned in the pJM1 suicidal vector and H37Rv genome. (**B**) *sufR*_*TB*_ (Rv1460) gene replaced by the hygromycin cassette in the H37Rv genome due to homologous recombination, generating a deletion mutant. (**C**) For Southern blot analysis, genomic DNA was isolated from different strains by using the CTAB method. DNA (5 µg) was digested with NcoI and transferred onto nitrocellulose membranes and probed with a DIG-labelled specific probe, upstream to *sufR*_*TB*_ gene. (**D**) Quantitative PCR depicting the upregulation of the ISC operon in the mutant strain grown in 7H9 medium enriched with 10% OADC and 0.05% Tween 80.

Unlike the six-gene operon in *Escherichia coli* and the suf locus *sufABCDSE* in *Erwinia chrysanthemi*^20^, the ISC operon in Mtb is a seven-gene operon *sufRBDCS (rv1460–66)*, and the orthologs of *sufA* and *sufE* are not present in Mtb. In addition, the *rv1466* gene of the ISC operon in Mtb is not homologous to any of the ISC genes and is predicted to have metal–sulphur cluster biosynthetic enzymes^21^ (Fig. S1). The ISC operon in mycobacteria is fairly conserved across species with the exception of the presence of an intein-invading sequence at two different sites of *sufB* gene in Mtb and *M. leprae*^21–23^. To study the contribution of *sufR*_*TB*_ gene, we employed a reverse genetic approach and generated an *sufR*_*TB*_ knockout strain of Mtb through homologous recombination (Fig. 1A). In this process, the *sufR*_*TB*_ gene of Mtb was disrupted by the insertion of a hygromycin marker cassette within the coding region^24^ (Fig. 1B). The formation of the mutant strain was confirmed through polymerase chain reaction (PCR) (Fig. S3A) by using primers amplifying the hygromycin cassette and flanking regions (Table S1) and through Southern blotting (Fig. 1C). Furthermore, the findings of semi-quantitative reverse transcription (RT)-PCR revealed the absence of the *sufR*_*TB*_ gene transcript, confirming its deletion in the mutant strain (Fig. S3B). We also found that Fe–S cluster genes were constitutively upregulated in *ΔsufR*_*TB*_ (Fig. 1D), indicating that SufR_TB_ protein might negatively regulate the ISC operon in Mtb.

ISC genes are organized as an operon in organisms such as *E. chrysanthemi*, *E. coli*, and *M. smegmatis*^21,25–27^. We designed an experiment to confirm the same in Mtb. Briefly, cDNA prepared from the Mtb culture was used as a template to amplify the gene fragment overlapping the junction regions of all genes belonging to the ISC locus (Fig. S2A). The amplification of the junction region demonstrated that all seven genes are cotranscribed as a single mRNA transcript and organized as an operon (Fig. S2B).

### *sufR*_*TB*_ gene of Mtb is essential for growth under a low-Fe condition

**Figure 2:**
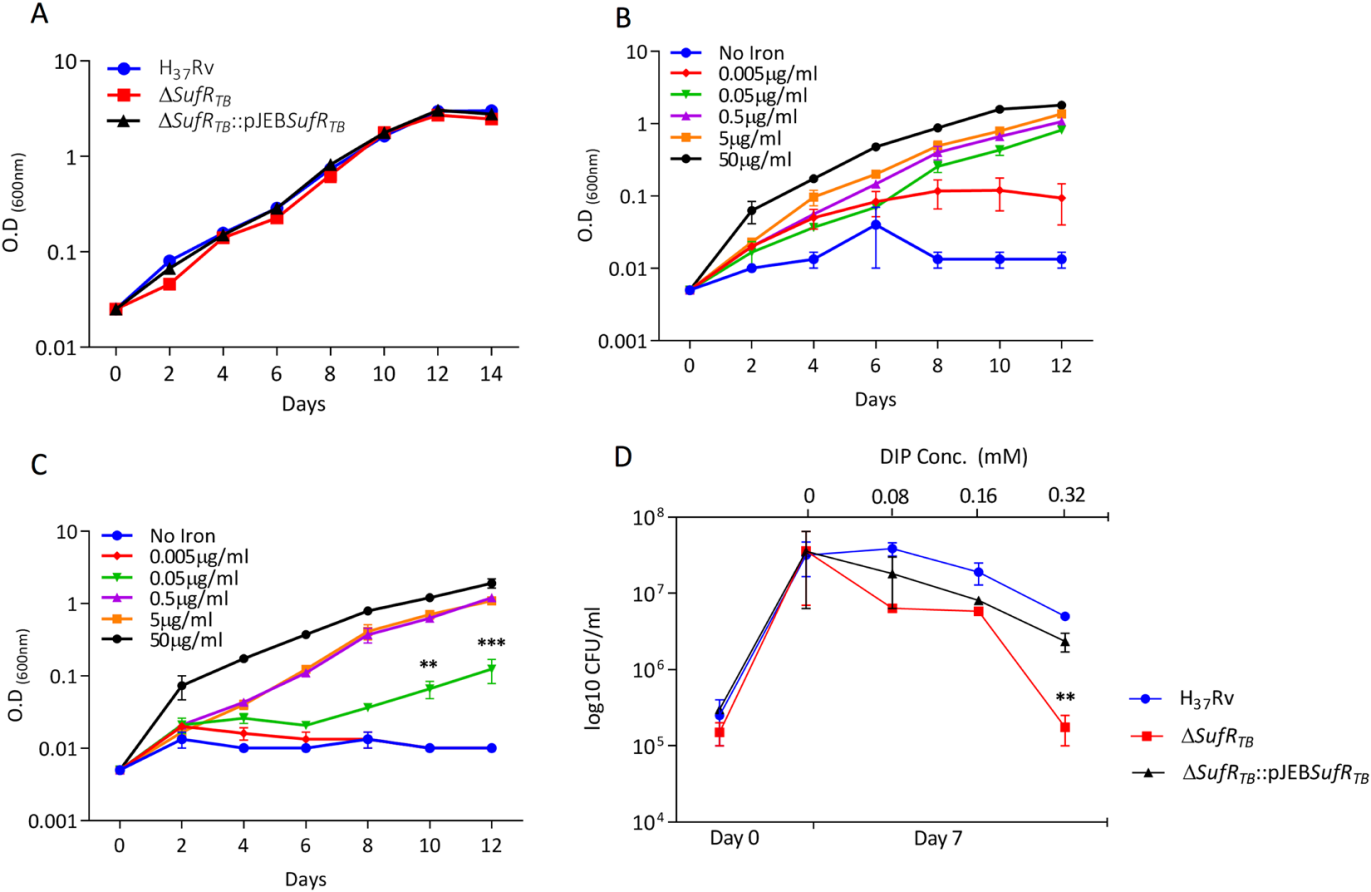
∆ *sufR*_*TB*_ fails to grow under a low iron condition. (**A**) Growth curve of wild-type, mutant, and complemented strains in 7H9 enriched medium. (**B** and **C**) Growth curve of wild-type (H37Rv) and ∆ *sufR*_*TB*_ strains in minimal medium supplemented with 0.1% glycerol at varying concentrations of ferric ammonium citrate. (**D**) Growth analysis of wild-type, mutant, and complemented strains in 7H9 enriched medium containing different concentrations of the Fe chelator, depicting growth attenuation in the mutant strain treated with 0.32 mM of 2, 2′-bipyridyl.

The role of SufR protein as a transcriptional repressor of ISC operon genes in both cyanobacteria^28^ and Mtb^15^ is well-documented. In addition, compared with the wild-type strain, the *ΔsufR* null strain of cyanobacteria demonstrated a higher growth rate under a low-Fe condition^27^. Expecting a similar phenotype, we examined the growth of *ΔsufR*_*TB*_ under a low-Fe condition. As a control, we first confirmed whether the growth rate differed between the wild-type strain and *ΔsufR*_*TB*_ strain grown in an enriched medium under an Fe-replete condition (Fig. 2A). However, in contrast to cyanobacteria, *ΔsufR*_*TB*_ showed a growth defect phenotype under a low-Fe condition. Compared with the wild-type strain (Fig. 2B), the mutant strain (Fig. 2C) required a 10-fold higher concentration of Fe for its growth. Furthermore, the addition of an Fe-chelating agent, 2, 2′-bipyridyl, adversely affected the growth of *ΔsufR*_*TB*_, highlighting the role of SufR_TB_ protein in sensing and regulating the growth of Mtb under a low-Fe condition (Fig. 2D). The possibility of any downstream polar effect caused by insertional inactivation was ruled out because a similar phenotype was observed in an unmarked *ΔsufR*_*TB*_ strain in which the insertion sequence was floxed out using the Cre recombinase system (Supplementary Fig. 3C). These findings suggest that the downregulation of ISC operon genes is essential for the growth of Mtb under iron-limiting conditions.

### *SufR*_*TB*_ promotes growth of Mtb under stress and in mouse BMDMs

**Figure 3:**
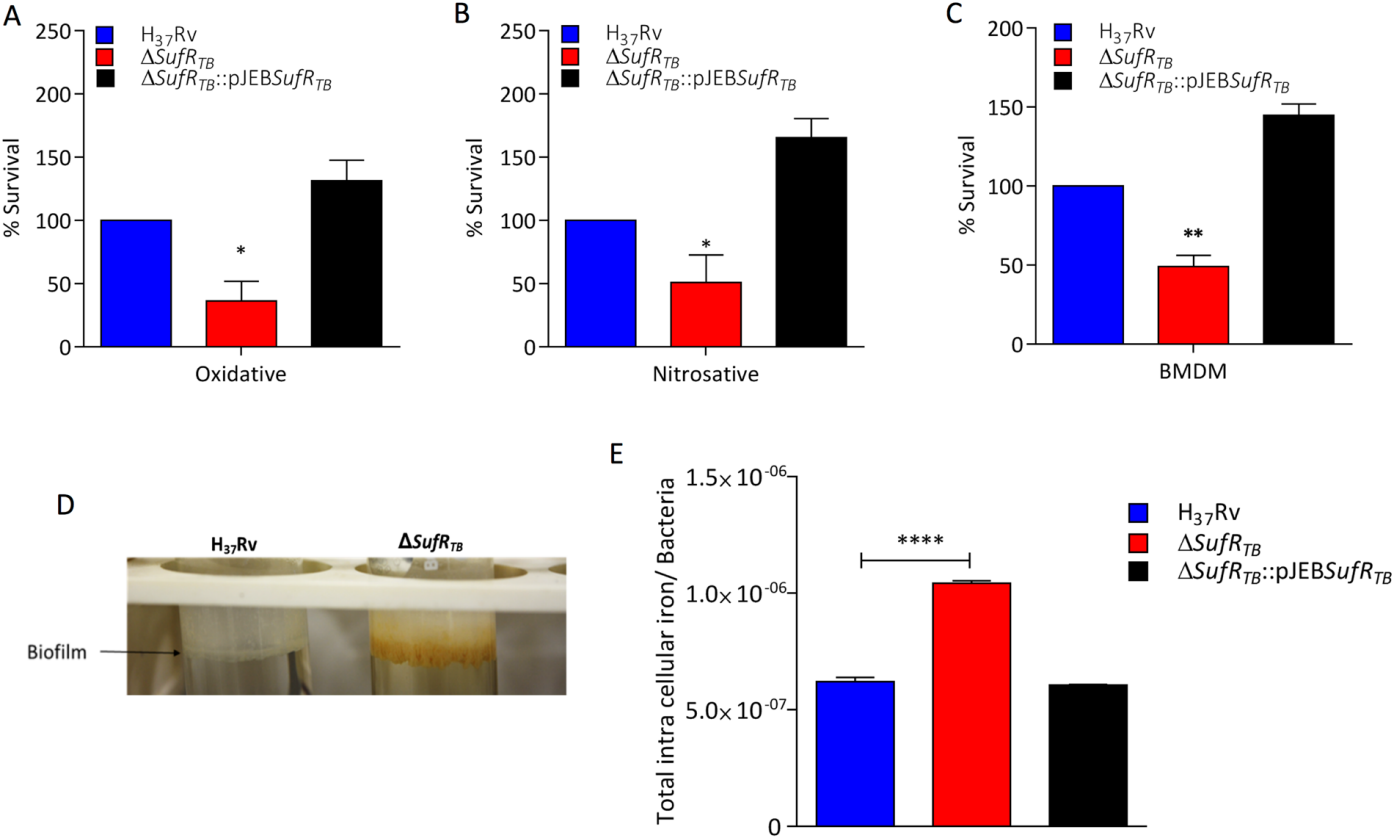
SufR_TB_ protein is essential for Mtb to survive under stress conditions. (**A** and **B**) Survival of wild-type, mutant, and complemented strains in 7H9 enriched medium under oxidative (5 mM H_2_O_2_ for 6 hours) and nitrosative stress (200 μM DETA-NO for 48 hours), respectively. (**C)** Growth of the ∆ *sufR*_*TB*_ strain relative to the wild-type strain in mouse bone marrow-derived macrophages. Macrophages were infected at an MOI of 1, and a relative growth difference was estimated by counting colony-forming units at day 0 and day 7 after plating. (**D**) Biofilm formation was observed in wild-type and mutant strains in Sauton’s medium over a period of 4 weeks. Significant differences observed in the groups are marked (unpaired two-tailed t test, *P < 0.005). (**E**) Estimation of intracellular Fe accumulated in H37Rv, ∆ *sufR*_*TB*_, and ∆ *sufR*_*TB*_:pJEB *sufR*_*TB*_ strains.

Mtb survives and favourably replicates inside macrophages, which are recruited by the host to eliminate the pathogen^29^. Mtb rapidly adapts to an extremely hostile, nutrient-limited environment by activating pathways that help neutralize host-mediated oxidative and nitrosative stress^30^. A majority of these pathway enzymes require Fe–S as a cofactor. Because SufR_TB_ protein represses the expression of genes critical for Fe–S biogenesis, we assessed the role of this protein in abrogating the host-mediated redox assault. We first confirmed whether the sensitivity of *ΔsufR*_*TB*_ towards SDS and antibiotics was similar to that of the wild-type strain (Supplementary Fig. 4A,B). Furthermore, we analyzed the relative sensitivity of *ΔsufR*_*TB*_ to free radicals by exposing both wild-type and *ΔsufR*_*TB*_ strains to H_2_O_2_ and nitric oxide (NO) at different concentrations and times. We found that compared with the wild-type Mtb strain, the *sufR*_*TB*_ null strain was more sensitive when exposed to both oxidative (Fig. 3A) and nitrosative stress (Fig. 3B). In addition, *ΔsufR*_*TB*_ demonstrated an enhanced biofilm production phenotype, and the produced biofilm was brown in color (Fig. 3D). These findings might be attributed to an increase in the concentrations of Fe-sequestering ISC operon proteins. To verify this, we quantified the total intracellular Fe level in different strains through inductively coupled plasma mass spectrometry (ICP-MS). As expected, compared with the wild-type strain, *ΔsufR*_*TB*_ had a two-fold higher level of intracellular Fe. The intracellular Fe level was restored to the wild-type level in the complemented strain, further implicating the role of SufR_TB_ protein in regulating Fe homeostasis in Mtb (Fig. 3E).

Because Fe sequestration is one of the host defense strategies to starve the pathogen of Fe, we quantified the relative ability of *ΔsufR*_*TB*_ and wild-type Mtb strains to survive in an Fe-limiting environment inside macrophages. Briefly, mouse BMDMs were infected at a multiplicity of infection (MOI) of 1, followed by quantifying their relative growth by plating and enumerating the colony-forming units (CFUs) 7 days post infection. As expected, 7 days post infection, relative to the wild-type strain, the *ΔsufR*_*TB*_ strain demonstrated a 50% reduction in its ability to survive in mouse BMDMs (Fig. 3C). As a control, we confirmed that the deletion of *sufR*_*TB*_ gene had no effect on the uptake of the pathogen by BMDMs (Fig. S5A).

### *SufR*_*TB*_ gene is critical for survival of Mtb inside the host

**Figure 4:**
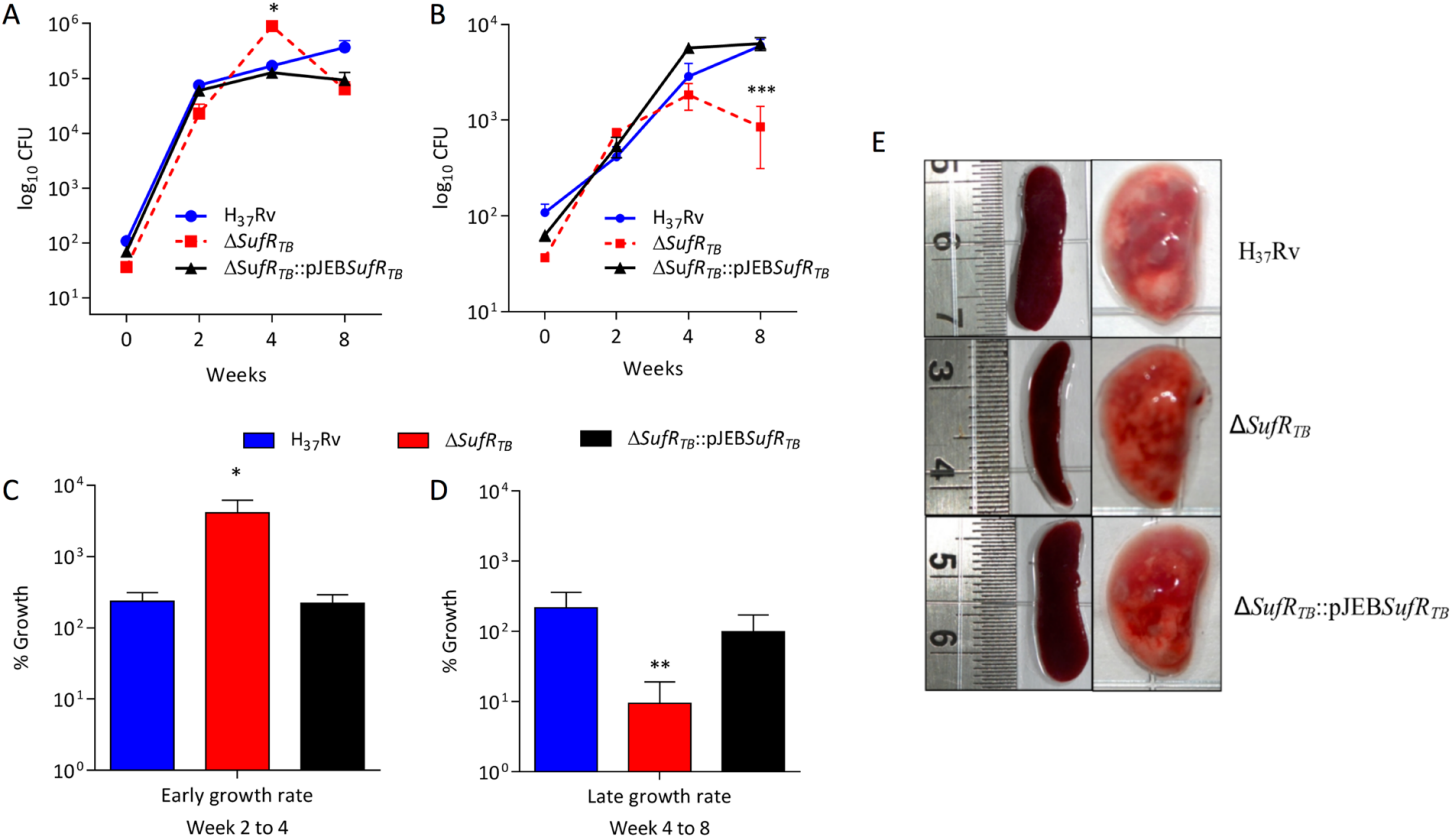
∆*sufRTB* gene is critical for survival of Mtb in the host. (**A** and **B**) Bacterial load in mice infected with different Mtb strains through the aerosol route (H37Rv, ∆*sufR*_*TB*_, and ∆*sufR*_*TB*_:pJEB *sufR*_*TB*_) in the lung and spleen, respectively, at week 2, 4, and 8 post infection. The data represents the average colony-forming count from 5 animals in each group. (**C** and **D**) The graph depicts the difference in the growth rate of different strains of Mtb in mice at early (2–4 weeks) and late (4–8 weeks) stages of Mtb infection. (**E**) Gross pathology of the lungs and spleens of animals infected with various strains of Mtb at 8 weeks post infection. Significant differences observed in the groups are marked (unpaired two-tailed t test, *P < 0.005)

Our data clearly indicate the essentiality of *sufR*_*TB*_ gene in regulating Mtb growth under a low-Fe condition. Furthermore, to evaluate the role of SufR_TB_ protein in the outcome of the disease process, we established an animal model by infecting mice with wild-type H37Rv, *ΔsufR*_*TB*_, and *ΔsufR*_*TB*_::pJEB*sufR*_*TB*_ strains. Briefly, 8–10-week-old C57/BL6 mice were infected with the aforementioned strains through the aerosol route, and the *in vivo* growth of these strains was evaluated by counting CFUs formed after plating the homogenates of the lungs and spleens harvested from mice at different time points post infection. Four weeks post infection, the ΔsufRTB strain was found to be growing at a relatively faster rate than did the wild-type strain. Unlike the wild-type strain, the mutant strain failed to persist and showed a pronounced growth defect 8 weeks post infection in both the lungs (Fig. 4A and C) and spleens (Fig. 4B and D). On gross examination 8 weeks post infection, it was found that the lungs of mice infected with the *ΔsufR*_*TB*_ strain had fewer visual surface granuloma than did the lungs of mice infected with the wild-type strain (Fig. 4E). Similarly, enlargement of the spleen was observed in mice infected with the wild-type strain but not in mice infected with the *ΔsufR*_*TB*_ strain. All the observed mutant-specific phenotypes were restored to wild-type levels in mice infected with the complemented strain, confirming the gene-specific effect.

## Discussion

The current study investigated the role of SufR_TB_ protein in the regulation of the ISC operon and its implications on mycobacterial pathogenesis. We found that similar to *M. smegmatis*^21^, ISC cluster genes in Mtb were transcribed as a single gene transcript and organized as an operon. The first gene of the operon, *sufR*_*TB*_ (*rv1460*), a Zn-finger domain-containing protein, inversely regulates the expression of downstream ISC operon genes. The essentiality of *sufR*_*TB*_ gene in the growth of Mtb under a low-Fe condition and the enhanced accumulation of intracellular Fe in the *ΔsufR*_*TB*_ strain together suggest a critical role of SufR_TB_ protein in regulating Fe homeostasis in Mtb. Disruption of Fe homeostasis caused a reduction in the growth of the *ΔsufR*_*TB*_ strain in mouse BMDMs. This transcription repressor protein was also required for the growth of Mtb under oxidative and nitrosative stress conditions. The enhanced biofilm production phenotype observed in *ΔsufR*_*TB*_ is intriguing and suggests an Fe-dependent regulation of biofilm generation in mycobacteria. Finally, we demonstrated that SufR_TB_ protein-mediated regulation of Fe homeostasis is required for mycobacterial persistence. Obtaining mechanistic insights into the effect of stress and Fe concentrations on the allosteric regulation of the ISC operon by SufR_TB_ protein can be an interesting area for future research.

The results of RT-PCR demonstrated that suf operon genes in Mtb are transcribed as a single mRNA transcript, suggesting that these genes are organized as an operon. While our work was under revision, a study published by another group reported a similar finding, confirming the role of SufR_TB_ protein as a repressor of sufR operon genes^15^. This finding is in line with those of several studies of other bacterial species that demonstrated sufR homologues to be a repressor of the suf operon across several bacterial species^27,28^.

Although Fe is essential, excess accumulation of intracellular free Fe is toxic^31^. Failure to regulate the intracellular Fe level might cause death either due to Fe deficiency or toxicity^32,33^. Because Fe deprivation is one of the antimicrobial strategies that the host adopts^33^, both the pathogen and host compete for limited Fe during infection^17^. In contrast to the finding of a recent publication^15^, we demonstrated that SufR_TB_ protein is essential for the growth of Mtb under a low-Fe condition. This discrepancy in findings may be because a pure *sufR*_*TB*_ gene deletion mutant strain could not be generated in the previous study, which might have led to inaccurate results. Disruption of the *sufR* homologue in cyanobacteria^27^ resulted in a decreased sensitivity to the Fe chelator 2, 2′-bipyridyl and an increased growth rate under a low-Fe condition. In cyanobacteria, this could possibly be due to a mechanistic difference in the regulation of the ISC operon. Moreover, the *sufR* gene homologue of cyanobacteria has a reverse orientation, implying a possible difference in its regulatory network. Mtb contains 20 enzymes belonging to the cytochrome P450 family that significantly contributes to its growth and requires the Fe–S cluster as a cofactor^34^. The low dependence of cyanobacteria on these enzymes could possibly lead to differences in their sensitivity to low Fe concentrations. We believe a single-crossover strain used in *M. smegmatis* study had an insertion at the 5′ end of *rv1461* gene with both *sufR* gene and the downstream ISC operon completely intact. The data observed may have been independent of SufR protein and could be due to the polar effect observed as a result of the insertion of a DNA sequence upstream to the *rv1461* gene of the ISC operon.

The susceptibility of the *ΔsufR*_*TB*_ strain to low Fe and its decreased ability to replicate in mouse BMDMs could be due to the presence of excess Fe-mobilizing ISC proteins; this was substantiated by quantifying the intracellular concentration of total Fe. Because Mtb encounters an extremely hostile, Fe-limiting condition in the host^35^, we believe that sequestration of excess Fe by ISC proteins in *ΔsufR*_*TB*_ is detrimental for its intracellular survival. Under a low-Fe condition, an excess of Fe-binding proteins sequesters most of the available Fe blocking the synthesis of the Fe–S cofactor. This results in a decrease in the activity of Fe–S cofactor–dependent enzymes critical for growth. Similarly, susceptibility of *ΔsufR*_*TB*_ to oxidative and nitrosative stress could be attributed to the excess accumulation of Fe-bound ISC proteins in the *sufR*_*TB*_ mutant. Under stress, these Fe-bound proteins release excess of free Fe, triggering the Fenton reaction and subsequently causing cell death^36^. In addition, the increased intracellular Fe level favouring biofilm formation in the mutant strain was not surprising. The role of Fe in biofilm production is well-documented in several bacterial pathogens including Mtb^37^, *Pseudomonas aeruginosa*^38^, *Staphylococcus aureus*^39^, and *Campylobacter jejuni*^40^.

The inability of *ΔsufR*_*TB*_ to persist in the host was very intriguing. Our study results suggest that during the early stage of Mtb infection, active recycling of transferrin receptors through the endocytic network ensures an Fe-replete condition in host phagosomes, resulting in a higher replication rate of *ΔsufR*_*TB*_. Because the onset of adaptive immunity inhibits transferrin receptor-mediated uptake of Fe by macrophages, there is an Fe-limiting condition generated in cells^41^. As a corrective measure, through SufR_TB_ protein, Mtb downregulates Fe-mobilizing ISC proteins, thereby restoring intracellular Fe homeostasis and facilitating long-term persistence in the host. Failure to do so in the *sufR*_*TB*_ gene mutant leads to a persistent growth defect phenotype.

Understanding Fe sensing through redox biology and its implication on the binding affinity of the SufR_TB_ repressor to its promoter can be a subject for future research. Our data suggest that Mtb ensures the availability and uniform distribution of Fe during growth under an Fe-limiting condition. Mtb achieves this by tightly regulating the transcript levels of gene-encoding enzymes that sequester and store Fe. We identified an ISC repressor protein, SufR_TB_, responsible for maintaining intracellular Fe homeostasis in Mtb. Under Fe-limiting conditions, SufR_TB_ protein downregulates genes encoding proteins that are responsible for sequestering and mobilizing Fe during the synthesis of the Fe–S cluster. This ensures the availability of Fe as a cofactor for enzymes essential for Mtb to grow under a low-Fe condition. We also demonstrated that maintenance of Fe homeostasis through SufR_TB_ protein is essential for Mtb to persist in the host. Furthermore, inhibiting the activity of SufR_TB_ protein through the identification of small molecule inhibitors can possibly be used as a novel intervention strategy to target persister populations during tuberculosis infection.

## Material and Methods

### Bacterial strains and culture conditions

All mycobacterial strains were grown and maintained in Middlebrook 7H9 broth and 7H11 agar (BD Difco cat# 271310 and 283810) supplemented with 10% OADS (bovine serum albumin, oleic acid, and dextrose); 0.05% Tween was added in 7H9 broth to enhance bacterial growth. All *E. coli* strains were grown in Luria–Bertani (LB) medium (BD Difco cat no. 244620). To achieve low-Fe growth conditions, strains were grown in a minimal medium containing 0.5 g/L of asparagine, 1 g/L of KH_2_PO_4_, 2.5 g/L of Na_2_HPO_4_, varying concentrations of ferric ammonium citrate, 0.5 g/L of MgSO_4_·7 H_2_O, 0.5 mg/L of CaCl_2_, 0.1 mg/L of ZnSO_4_, and 0.5 mg/L of tyloxapol. Growth was examined by measuring absorbance at an optical density of 600 nm and by plating on 7H11 agar plates. The concentrations of antibiotics used were as follows: kanamycin (100 µg/mL for *E. coli* and 25 µg/mL for Mtb) and hygromycin B (150 µg/mL for *E. coli* and 50 µg/mL for Mtb).

### Quantitative and semi-quantitative PCR

For RT and qRT PCR studies, mid-log-phase cultures of various Mtb strains grown in 7H9 medium were transferred into an Fe-free medium for 48 hours for Fe starvation. After 48 hours, the cultures were resuspended in a medium containing 0.05 µg/mL of Fe. RNA isolation was performed using the Qiagen RNeasy mini kit (cat # 74104), and DNase treatment was performed using the Turbo DNA free kit (Thermo Fischer Scientific, Cat# AM1907). cDNA was prepared using the AccuScript High Fidelity 1st Strand cDNA synthesis kit (Agilent, cat# 200820) according to the manufacturer’s protocol. For operon analysis, primers designed for intergenic regions were used for the semi-quantitative analysis of the transcript through RT-PCR. For qRT-PCR, the threshold cycle (CT) value obtained for each gene was normalized with the value obtained for the housekeeping gene (*sigA*) to obtain ∆Ct values. The transcript levels of ISC operon genes were measured under normal and low-Fe conditions in wild-type H37Rv and *∆sufR*_*TB*_ strains. Primers used in this study are listed in Table S2.

### Construction of *∆sufR*_*TB*_ and *∆sufR*_*TB*_: pJEB*sufR*_*TB*_ strains

The deletion of *sufR*_*TB*_ gene in the H37Rv strain of Mtb was performed using the site-specific homologous recombination strategy. Briefly, after cloning the 1000-bp flanking regions of the gene by using F1-R1 and F2-R2 primer pairs (Table S1) in the pJM1 vector, the construct was electroporated into Mtb electrocompetent cells. A double-crossover mutant was screened after a two-step recombination event. The mutant strain was further confirmed using a PCR-based screening strategy. Disruption of ∆ *sufR*_*TB*_ gene was substantiated by southern blot analysis. DNA isolation of H37Rv and ∆*sufR*_*TB*_ mutant strains were performed using CTAB method^42^. 5 µg of DNA was digested with Nco I restriction enzyme and transferred on Hybond Nitrocellulose membrane (mdi membrane technologies, cat# SNPZ8302XXXX101). A 537 bp sequence region upstream of *sufR* gene was used as a probe for the identification of sequence. Probe was DIG labelled using DNA Labeling and Detection Kit (Merck cat#11585614910) as per the manufacturer’s protocol. The complementation strain was generated by adding back a copy of *sufR*_*TB*_ gene cloned into the pJEB402 integrative vector in the ∆*sufR*_*TB*_ mutant strain.

### Stress experiments

Wild-type, *∆sufR*_*TB*_, and *∆sufR*_*TB*_:pJEB*∆sufR*_*TB*_ strains of Mtb were grown in Middlebrook 7H9 supplemented medium. Mid-log-phase cultures were washed with 7H9 medium and exposed to 5 mM H_2_O_2_ (ROS) for 6 hours and 200 µM NO adduct, DETA-NO (RNS), for 48 hours. DETA-NO was replenished after 24 hours. To examine the cell wall integrity of the strains, the cultures were exposed to an SDS surfactant containing 0.1% SDS. To examine drug sensitivity, strains were exposed to the following antituberculosis drugs: 0.0625 µg/mL of rifampicin (a replication inhibitor), 0.125 µg/mL of isoniazid (an inhibitor of mycolic acid synthesis), 3 µg/mL of kanamycin (a translational inhibitor), and 0.5 µg/mL of moxifloxacin (a DNA gyrase inhibitor). Survival was examined by counting CFUs on agar plates at different time points. To study growth kinetics under low-Fe conditions, mid-log-phase cultures were exposed to different concentrations of the Fe chelator 2, 2′-bipyridyl (Sigma, cat # D216305). Growth was analyzed by counting CFUs on 7H11 agar plates at various time points. To investigate the effect of different Fe concentrations, mid-log-phase cultures were washed with phosphate-buffered saline (PBS) and transferred into an Fe-free media for 48 hours, after which they were again transferred into a chelated minimal medium containing different Fe concentrations (0.005, 0.05, 0.5, 5, and 50 µg/mL). Growth was analyzed by measuring absorbance at 600 nm at different time points.

### Biofilm experiments

Biofilm formation experiments were performed using Sauton’s medium. Log-phase cultures of various Mtb strains were washed with PBS, and 200 µL of the cultures were transferred into glass tubes containing 20 mL of the medium. The tubes were sealed and incubated at 37 °C for 22 days. After 3 weeks, the caps of the tubes were relaxed and biofilm formation was observed according to the standard protocol^43^.

### *In-vitro* growth assay using BMDMs

C57Bl/6 mice were sacrificed, and BMDMs were harvested and cultured in DMEM medium supplemented with 10% FBS and the L929 cell line supernatant. After maturation, 5 × 10^5^ cells were seeded in a 24-well plate and infected with different Mtb strains with an MOI of 1. After 4 hours of infection, cells were washed with 1 × PBS (Gibco, Thermo Fischer) thrice to remove extracellular bacteria. Subsequently, cells were lysed with 0.1% Triton X-100 at different time points. The intracellular growth of different strains was measured by plating the lysate at various dilutions and counting CFUs.

### Intracellular Fe estimation

Strains were grown under a standard Fe condition, and cultures with an optical density of 0.7–0.8 were pelleted down and resuspended in the buffer. Then, 100 µL of the sample was removed from the provided sample vials^44,45^, and mixed with 9.9 mL of miliQ water to prepare a 100 times dilution of the sample. Next, these diluted samples were analyzed through ICP-MS; during the analysis, 3 mL of the 10-mL sample was consumed by sample injector channels. The general setup configuration of ICP-MS (Thermo Scientific Q-ICP-MS XSeries2) and the standard operating conditions used for the ICP-MS analysis is enlisted in Table S4.

### Mouse infection study

Animal experiment protocols were reviewed and approved by the Institutional Animal Ethics Committee of International Centre for Genetic Engineering and Biotechnology, New Delhi, India (ICGEB/AH/2015/TACG-THSTI-9). Animal experiments were performed in accordance with guidelines provided by the Committee for the Purpose of Control and Supervision of Experiments on Animals (Govt. of India). Pathogen-free C57BL/6 mice were obtained from the National Centre for Laboratory and Animal Science, Telangana, India. Eight-week-old C57BL/6 mice were infected through aerosol exposure in the Madison Aerosol Chamber with approximately 100 CFUs of different strains (H37Rv, *∆sufR*_*TB*_, and *∆sufR*_*TB*_: pJEB*∆sufR*_*TB*_). Mice were infected with different Mtb strains through aerosol route. Mice from each group (N = 5) were sacrificed at different time points (Day 0, 2, and 4 and 8 weeks post infection). Their lungs and spleen were removed, and the serial dilution of the organ homogenate was plated on 7H11 agar plates supplemented with 10% OADS for bacterial enumeration.

## Electronic supplementary material

Supplementary information
