# Supplementary material for: Iron homeostasis in Mycobacterium tuberculosis is essential for persistence
Source: Sci Rep. 2018 Nov 26;8:17359. doi: 10.1038/s41598-018-35012-3 (PMC6255865; doi:10.1038/s41598-018-35012-3)
Supplement: Supplementary file 1 — Supplementary information [file 41598_2018_35012_MOESM1_ESM.pdf]

Title: Iron homeostasis in *Mycobacterium tuberculosis* is essential for persistence

Manitosh Pandey[1], Sakshi Talwar[1] , Sutapa Bose[2] and Amit Kumar Pandey[1*]

[1]Mycobacterial Pathogenesis Laboratory, Translational Health Science and Technology Institute (THSTI), Faridabad 121001, Haryana, India;

[2]Environmental Science Research Laboratory, Dept. of Earth Sciences, Indian Institute of Science Education and Research, Kolkata, Nadia, India.

[*]Correspondence and requests for materials should be addressed to A.K.P. (email: amitpandey@thsti.res.in)

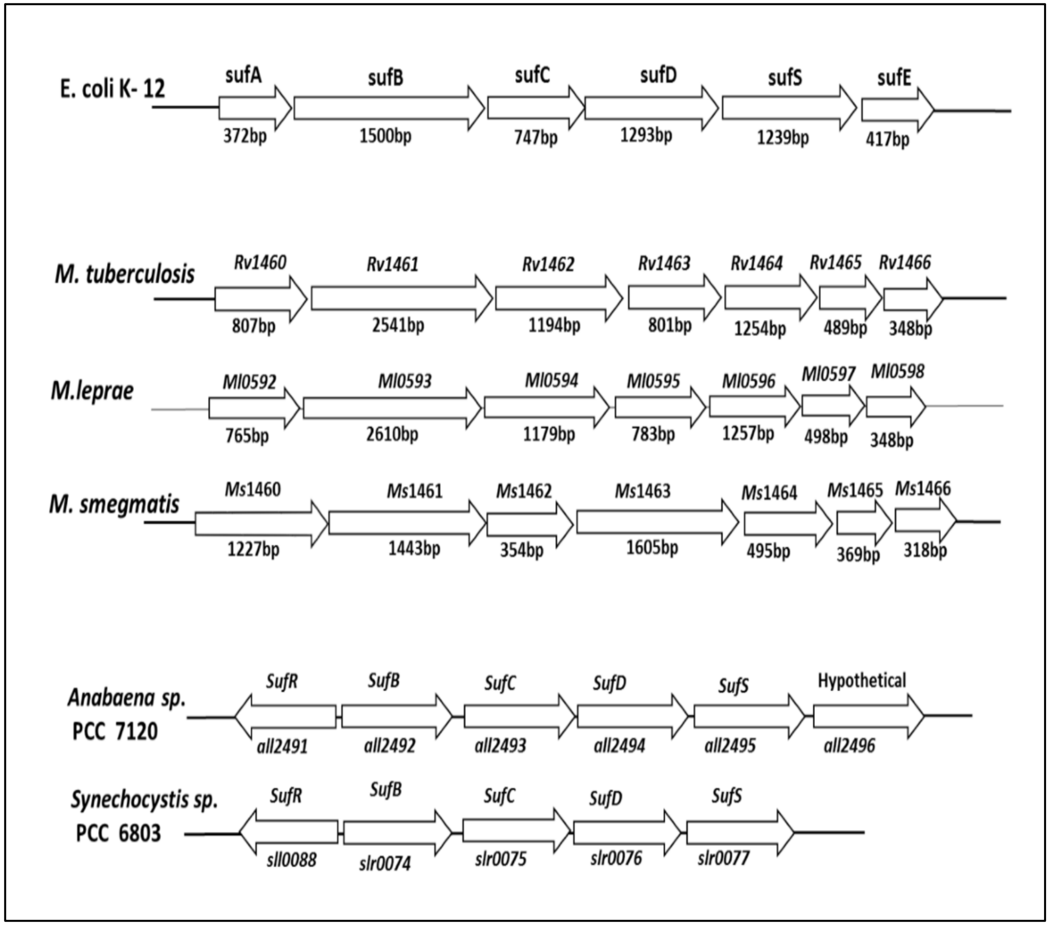

Supplementary Figure: 1

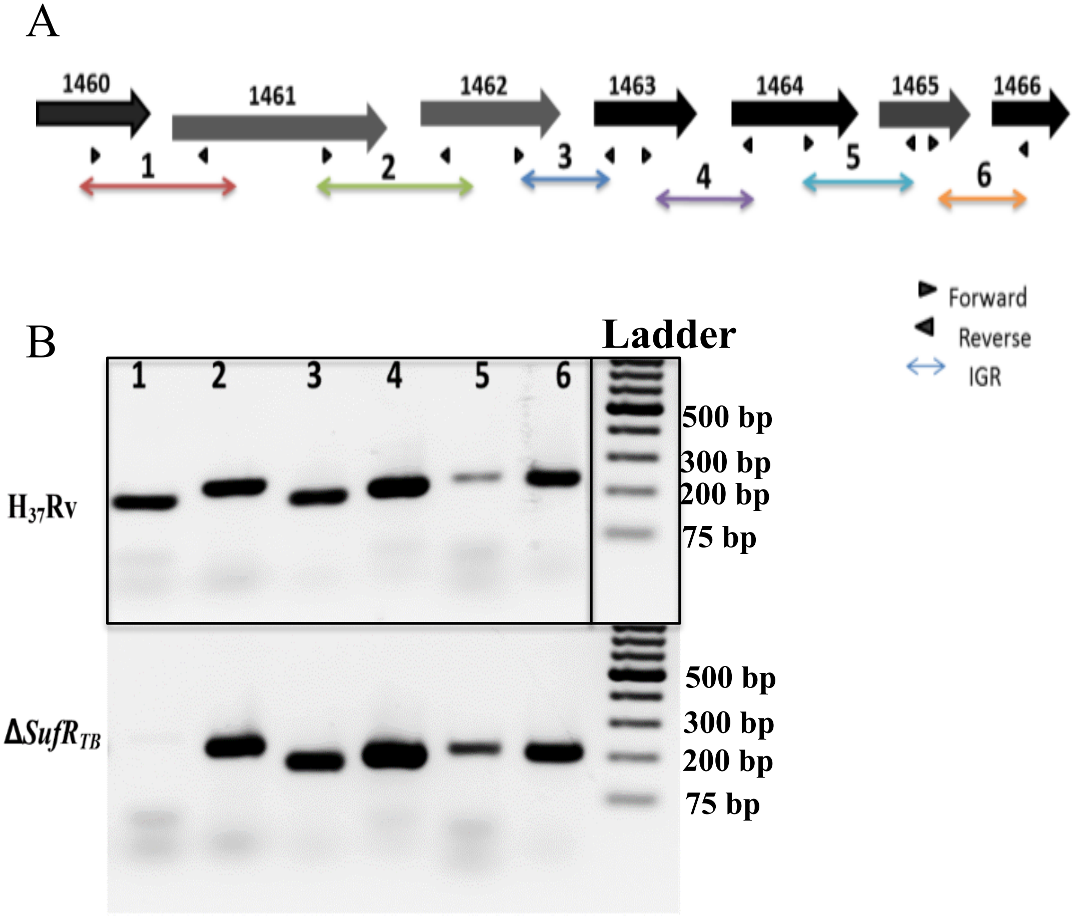

Supplementary Figure: 2

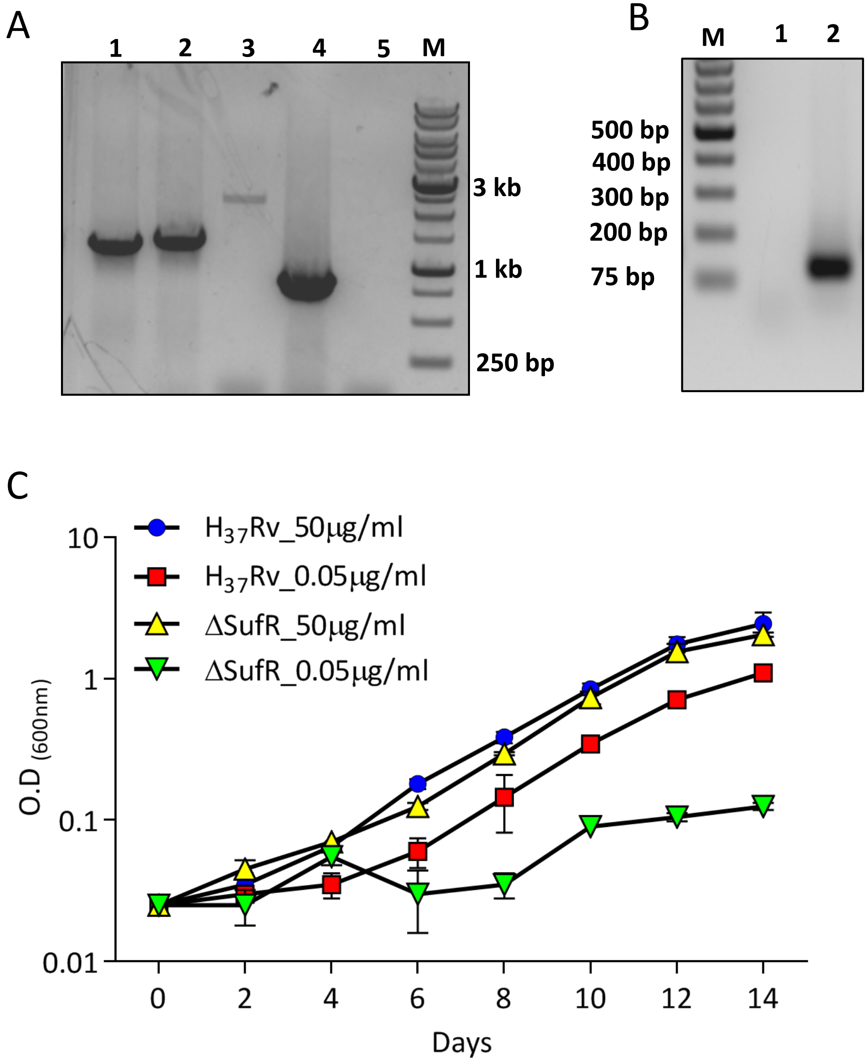

Supplementary Figure: 3

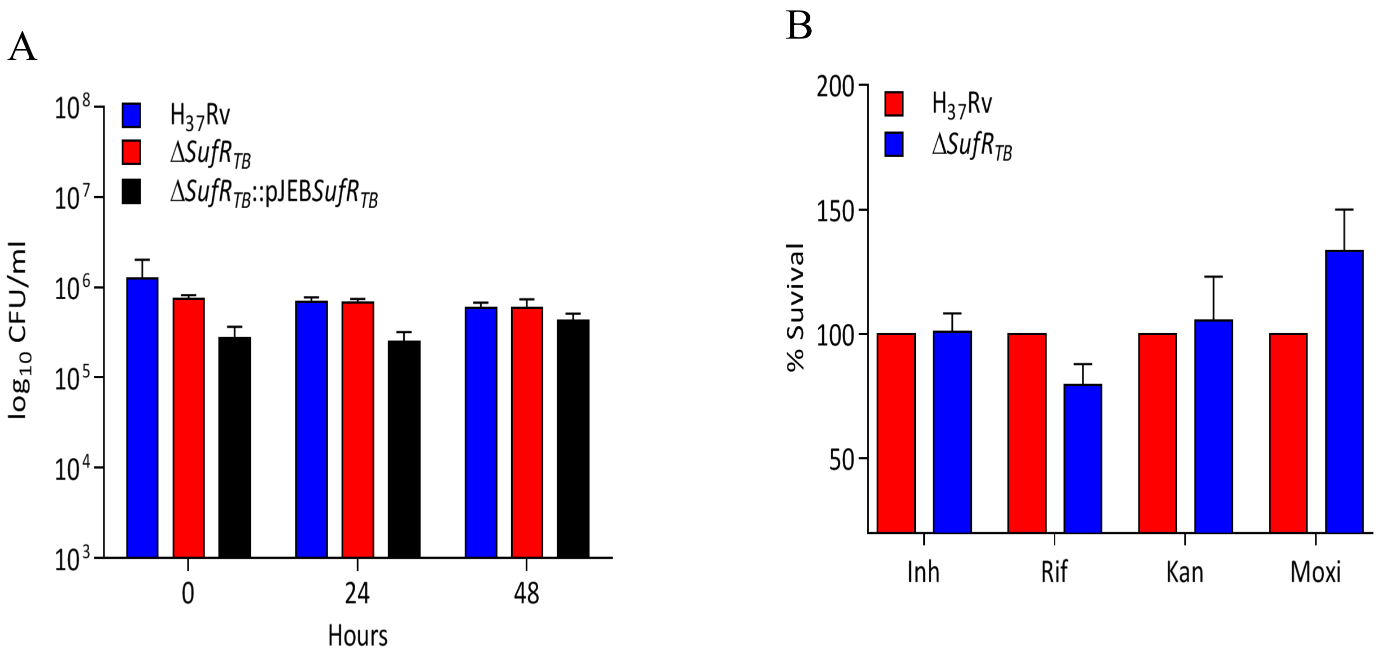

Supplementary Figure: 4

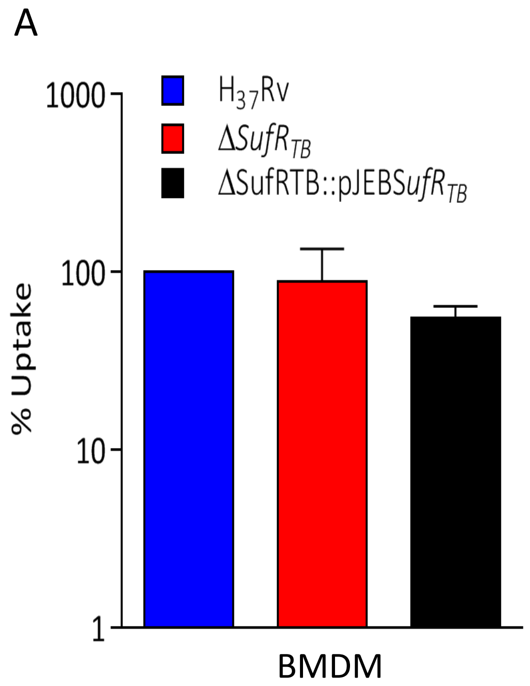

Supplementary Figure: 5

Supplementary figure Legends

Supplementary Figure 1: Map depicting comparative gene organization of the ISC locus in different bacteria. The gene organization of the ISC locus from three mycobacterial species were compared with the available sequence of *Synechocystis sp.* Strain PCC 6803, *Anabaena sp*. Strain PCC 7120 and the *E. coli* genome sequence. The arrows indicate the direction of the transcription.

Supplementary Figure 2: Transcript analysis of Suf operon in Mtb A. Schematic representation of ISC operon in Mtb, arrowheads show the primers used for the transcript analysis. **B.** PCR amplification of intergenic regions using cDNA from wild type and Δ *sufR*$_{TB}$ strains as a template.

Supplementary Figure 3: Mutant confirmation: A. *sufR*$_{TB}$ gene deletion mutant (Δ*sufR*$_{TB}$) confirmation by PCR. Lane1 & 2: Amplification of the *sufR* flanking region confirms gene specific insertion of the hygromycin cassette, Lane 3: 2.0 kb amplicon using *sufR*$_{TB}$ gene specific primers further confirms the insertional deletion of the *sufR*$_{TB}$ gene, lane 4: Depicts amplification of *sufR*$_{TB}$ gene (807bp) using H37Rv genomic DNA as a positive control, lane 5: No template control. **B.** The absence of *sufR* transcript in Δ *sufR*$_{TB}$ strains is further confirmed by semi-quantitative PCR using Δ *sufR*$_{TB}$ cDNA as a template (Lane 1) and wild type H37Rv (Lane 2) strains. **C.** Growth kinetics of unmarked *sufR*$_{TB}$ gene in minimal media at different iron concentrations.

Supplementary Figure 4: SDS and Drugs susceptibility assay: A. Survival of wild type, mutant and complemented strain in 7H9 enriched media containing 0.1% SDS. Growth differences were estimated by CFU plating at 24 and 48 hours post treatment. **B.** Survival of wild type, mutant and complemented strain in 7H9 enriched media containing 1X MIC of different anti TB drugs. Difference was estimated by CFU plating at 72 Hours.

Supplementary Figure 5: Drugs susceptibility: A. Survival of wild type, mutant and complemented strain in 7H9 enriched media with MIC of different anti TB drugs. Difference was estimated by CFU plating at 72 Hours.

Supplementary Figure 6: BMDM uptake assay: A. Relative uptake of H37Rv, Δ $sufR_{TB}$ and Δ $sufR_{TB}$:pJEB $sufR_{TB}$) in bone marrow derived macrophages, 4 hours post infection.

Table: S1
Mutant confirmation primers

Primer	Sequence
1460_F1	ATGATATCATTCCGGACGGGGTTTGACTG
1460_R1	ATGCGGCCGCTGATCGGTCTACCAGGGATGC
1460_F2	ATCTCGAGGGAGCGTCCCGATGACAC
1460_R2	ATGGGCCCTTCAGCCTTCCACCCATTGCG
Rv1460-conf1	CAGCGTGATCAGGATGGAACC
Rv1460-conf2	GGTAGATCAGGAAGTCACCCTC

Table: S2
Quantitative analysis primers

Primers	Sequence
1460-RT-F	ATCGCAACGGCGCTCAGCAAAG
1460-RT-R	CTGCTGCTCGGTTTCGCACAAT
1461-RT-F	CGTTCCAGTTGCAGTCCATCC
1461-RT-R	CACCTGGTAGATGTCCTTGCGAAC
1462-RT-F	CGACAACACCGTTCACCTCAGC
1462-RT-R	CGTCGTCGGCGAAATACAGC
1463-RT-F	CTGGGTCAAAGAGGTCAAGGC
1463-RT-R	GCGATCTTGGGCTTGAGCAG
1464-RT-F	GGCCCCGGCGACGTGATCGT
1464-RT-R	GTCCAGATACAGCGAGTCCAG
1465-RT-F	CACTACAAGCATCCGCAGCATC
1465-RT-R	ACAGCCTTGTCCGTCATAGGAAAC
1466-RT-F	GTGCAAGACGGTGACGAAGG
1466-RT-R	CCCAGTTGATGCGGATGTCGTC
1460/61-RT-F	CATCGTCAACGGAGACTGCG
1460/61-RT-R	ATCGCCTCTTCCTGGGTCAG
1461/62-RT-F	CTGTTCTACCTGATGAGCCGC
1461/62-RT-R	TCGCCCTTGTTGTGTGCGATC
1462/63-RT-F	CGCGGCTTCTTCGGTGAG
1462/63-RT-R	TCCGCGGGGTTCTCCAC
1463/64-RT-F	CGAACTCGACCAGAACGGC
1463/64-RT-R	CCGGAGTCCAAATACGCCAAC
1464/65-RT-F	GCGTCGTTCGCGGTGTAC
1464/65-RT-R	GCCGCAGATCGGGTTCAC
1465/66-RT-F	GTGAAATGCGCGCTGCTC
1465/66-RT-R	CGTCCAAGCCGTAGACCAG
SigA-RT-F	GAGGACCACGAAGCCTCGAAG
SigA-RT-R	GTTTGAGGTAGGCGCGAACC

Table: S3
Strains and Plasmids

Strains/Plasmids	Description	Source
XL-1 Blue (E. coli)	recA1 endA1 gyrA96 thi-1hsdR17 supE44 relA1 lac [F' proAB lacIq ZM15 Tn (Tetr)]	Stratagene, USA
$H_{37}Rv$	Laboratory strain of M.tb	A generous gift from Christopher M. Sassetti.
ΔsufR$_{TB}$ Rv1460 mutant	Mutant strain of $H_{37}Rv$	This study
ΔsufR$_{TB}$ (Unmarked strain)	Unmarked mutant strain of $H_{37}Rv$	This study
Δ*sufR*$_{TB}$:pJEBΔ*sufR*$_{TB}$Rv1460 complemented	Rv1460: kan complemented with Rv1460 at attB site.	This study
pJM1	Suicidal vector Hyg^R	A generous gift from Christopher M. Sassetti.
pJEB-402	Shuttle vector kan^K	A generous gift from Christopher M. Sassetti.

Table: S4
Standard operating conditions of the ICP-MS used for the analysis

PARAMETER	ICPMS (Thermo Scientific X-Series 2)
RF Power	4.2 Kw
Carrier Gas Flow Rate	20 ml/min
Collision Cell Gas	He (93%) + H (7%)
Collision Cell Gas Flow Rate	2-10 ml /min
Nebulizer Pump Rate	0.5 rps (30 RPM)
Uptake Time	30 s
Wash Time	60- 70 s
Standard Used (NIST 2711a)	2.82±0.04 (in %)
